# Diabetic cardiomyopathy as a disorder of proteostatic maladaptation: context-dependent ubiquitin-specific protease networks

**DOI:** 10.3389/fcell.2026.1910665

**Published:** 2026-09-18

**Authors:** Xiangyun Meng, Shuming Guo, Hui Lin, Ruchao Li, Wei Peng, Lingang Sun, Xiaowei Shi, Lixia Yang

**Affiliations:** 1 Department of Medical Laboratory Science, Gansu Provincial Hospital of Traditional Chinese Medicine, Lanzhou, Gansu, China; 2 Department of Intensive Care Unit, Gansu Provincial Hospital of Traditional Chinese Medicine, Lanzhou, Gansu, China; 3 Department of Medical Research Center, Shaoxing People’s Hospital, Zhejiang University Shaoxing Hospital, Shaoxing, Zhejiang, China; 4 School of Medicine, Shaoxing University, Shaoxing, Zhejiang, China; 5 Department of Endocrinology, Gansu Provincial Hospital of Traditional Chinese Medicine, Lanzhou, Gansu, China; 6 Gansu Academy of Chinese Medicine, Lanzhou, Gansu, China

**Keywords:** autophagy, deubiquitination, diabetic cardiomyopathy, ferroptosis, mitochondrial dysfunction, ubiquitin-specific protease

## Abstract

Diabetic cardiomyopathy (DCM) is a multidimensional myocardial remodeling disorder driven by interconnected metabolic, inflammatory, ferroptotic, autophagic and fibrotic programs, yet the post-translational mechanisms that convert diabetic stress into persistent injury remain incompletely integrated. Ubiquitin-specific proteases (USPs) regulate substrate stability, ubiquitin-chain architecture and signaling-complex dynamics and therefore represent an important proteostatic layer in DCM. Rather than assigning fixed protective or maladaptive identities to individual enzymes, this review organizes current evidence according to specific USP-substrate axes and their cellular and disease contexts. Predominantly protective axes include USP28-PPARα/Mfn2, USP8-Parkin, USP13-NLRP3, USP20-p62/STING, USP18-dependent stabilization of Notch1 or FOXC2, and the endothelial USP33-ATG7/FIS1 pathway. Predominantly injurious axes include USP38-ACAD11, USP14-VDAC1 and USP24-associated ferroptotic and autophagy-disruptive signaling. USP7 exemplifies substrate-dependent bidirectionality: USP7-PGC1β promotes metabolic dysfunction, whereas USP7-NRF2 supports GPX4-dependent ferroptosis resistance during empagliflozin treatment. By integrating these axes across pathological modules and cardiac cell types, this review reframes DCM as a disorder of context-dependent proteostatic maladaptation and highlights substrate-resolved USP regulation as a priority for future mechanistic and translational investigation.

## 1 Introduction

### 1.1 Diabetic cardiomyopathy: beyond metabolic overload

Diabetic cardiomyopathy (DCM) is increasingly recognized as a distinct myocardial complication of diabetes mellitus, characterized by structural and functional abnormalities of the heart that occur independently of overt coronary artery disease, hypertension, or valvular heart disease (Bugger and Abel, 2014; Jia et al., 2018; Dillmann, 2019; Tan et al., 2020). Clinically, DCM often begins with subtle myocardial stiffness, impaired relaxation, concentric remodeling, and diastolic dysfunction, and may gradually progress to systolic impairment, overt heart failure, and increased arrhythmogenic vulnerability (Borghetti et al., 2018; Huo et al., 2023). Because early diabetic myocardial remodeling may remain clinically silent for years, molecular injury can accumulate before conventional imaging or symptoms reveal established disease.

The conceptual framework of DCM has expanded beyond glucotoxicity, lipotoxicity, insulin resistance and altered substrate utilization. Current evidence indicates that DCM is a multidimensional remodeling syndrome involving mitochondrial dysfunction, oxidative stress, endoplasmic reticulum stress, sterile inflammation, cardiomyocyte death, microvascular rarefaction, impaired autophagy and progressive fibrosis (Frati et al., 2017; Gollmer et al., 2020; Huang et al., 2023; Bellemare et al., 2025). These processes interact in a self-reinforcing manner: mitochondrial injury increases reactive oxygen species and lipid peroxidation; oxidative stress activates inflammatory and profibrotic signaling; inflammatory cell death amplifies tissue remodeling; and fibrosis further compromises diastolic compliance and myocardial perfusion.

Although SGLT2 inhibitors and GLP-1 receptor agonists have improved cardiovascular management in patients with diabetes and heart failure risk, the residual burden of diabetic myocardial remodeling remains substantial (Yamada et al., 2021; Rolek et al., 2023; Tantawy et al., 2025). This suggests that additional molecular layers beyond systemic glucose lowering and hemodynamic modulation require attention. Among these, protein quality control and post-translational regulation are emerging as central mechanisms by which chronic diabetic stress is converted into persistent myocardial injury.

### 1.2 Protein homeostasis as an emerging layer in diabetic myocardial remodeling

The diabetic heart is exposed to a chronic biochemical environment characterized by hyperglycaemia, elevated fatty acids, advanced glycation end-products, oxidative stress and low-grade inflammation (Giraldo-Gonzalez et al., 2025; Han et al., 2026). These insults not only perturb metabolic flux but also reshape the myocardial proteome by affecting protein folding, stability, localization, complex formation and degradation. Protein homeostasis, or proteostasis, therefore represents a critical adaptive system that determines whether cardiomyocytes maintain functional resilience or progress toward maladaptive remodeling (Hussain, 2025).

The ubiquitin-proteasome system (UPS) is a principal machinery for selective protein degradation and protein quality control in eukaryotic cells (Swatek and Komander, 2016; Pohl and Dikic, 2019). In the heart, UPS activity contributes to removal of damaged, misfolded, oxidized or short-lived regulatory proteins, thereby preserving sarcomeric integrity, mitochondrial function, calcium handling and stress signaling (Su and Wang, 2010). Cardiac proteasome functional insufficiency has been implicated in cardiomyopathy and adverse remodeling, supporting the broader concept that many forms of cardiac disease may contain elements of cardiac proteinopathy (Su and Wang, 2010; Wang et al., 2011). In diabetes-induced myocardial injury, UPS components are dynamically altered and interact with other quality-control pathways such as autophagy (Li et al., 2017; Nahum-Ankonina et al., 2023).

Deubiquitinating enzymes (DUBs) counterbalance ubiquitination by removing or editing ubiquitin signals on substrate proteins. By regulating protein stability, ubiquitin-chain architecture and signaling-complex dynamics, DUBs can either amplify pathological signaling or preserve adaptive stress responses depending on substrate identity, cellular compartment and disease stage (Snyder and Silva, 2021; Trulsson et al., 2022). In DCM, this provides a mechanistic bridge between metabolic injury and downstream phenotypes such as mitochondrial dysfunction, inflammatory cell death, ferroptosis, autophagy impairment and fibrosis.

### 1.3 Conceptual framework of this review

The molecular landscape of USP biology in DCM has expanded rapidly, but most available studies remain organized around individual enzyme-substrate relationships. Although Li and Ma recently reviewed USPs as emerging regulators of cardiomyopathy more broadly, DCM has distinctive metabolic-inflammatory-proteostatic features that warrant a dedicated synthesis (Li and Ma, 2025). Importantly, the accumulating evidence does not support assigning an invariant protective or maladaptive identity to an entire USP enzyme. The biological outcome instead depends on the substrate being regulated, the cardiac cell type, the ubiquitin signal involved and the experimental or therapeutic context.

We therefore organize the evidence at the level of USP-substrate axes. Several axes exert predominantly protective effects in the reported setting, including USP28-PPARα/Mfn2 (Xie et al., 2024), USP8-Parkin (Sun et al., 2020), USP13-NLRP3 (Xu D. et al., 2026), USP20-p62/STING (Zhou et al., 2026), USP18-Notch1/FOXC2 (Cheng et al., 2025; Xu Z. et al., 2026), and USP33-ATG7/FIS1 (Chen et al., 2026). Other axes predominantly amplify diabetic myocardial injury, including USP38-ACAD11 (Xiao et al., 2025), USP14-VDAC1 (Wang et al., 2026) and USP24-associated ferroptotic and autophagy-disruptive signaling (Wu et al., 2024; Wu et al., 2026). USP7 provides the clearest example of bidirectional regulation: the USP7-PGC1β axis promotes maladaptive metabolic remodeling (Yan et al., 2024), whereas USP7-mediated NRF2 stabilization supports anti-ferroptotic protection during empagliflozin treatment (Cui et al., 2026). Accordingly, the terms “protective” and “injurious” are used throughout this review to describe the reported effect of a specific USP-substrate axis in a defined context rather than an intrinsic property of the USP enzyme. This axis-centered framework also allows direct non-ischemic DCM evidence to be distinguished from diabetic myocardial infarction, other cardiac injury models and mechanistically plausible extrapolations.

Within each module we further resolve USP function along two transversal axes. The first is cardiac cell type: the same enzyme can produce opposing outcomes in cardiomyocytes versus immune cells, as exemplified by USP13, which restrains NLRP3 inflammasome activity in cardiomyocytes (Xu D. et al., 2026) but stabilizes NLRP3 in macrophages by antagonizing TRIM31-mediated degradation (Li et al., 2025). The second is disease stage: different USP-substrate axes may become relevant as DCM progresses from metabolic remodeling to inflammatory amplification, cell death and fibrosis, although their temporal dominance has not been established longitudinally. Together, these dimensions establish that USPs are not peripheral modifiers but nodal regulators of diabetic myocardial remodeling whose function is shaped by substrate identity, ubiquitin-chain topology, cellular compartment and pathological context (Ren et al., 2023).

This integrative framework reframes DCM as a disorder of metabolic stress-driven proteostatic maladaptation in which USP-substrate axes function as nodal regulators rather than peripheral modifiers, and provides a framework for evaluating whether substrate- and cell type-resolved USP modulation may ultimately have translational relevance. The remainder of this review is organized accordingly: Sections 3 and 4 examine USP-substrate axes with predominantly protective, injurious or bidirectional effects in mechanistic detail, Section 5 considers underexplored candidates, Section 6 maps these enzymes onto five DCM pathological programs, Section 7 resolves USP function along the cell type and disease stage axes, and Sections 8 and 9 translate the framework into therapeutic and translational priorities. This conceptual framework is depicted in Figure 1.

**FIGURE 1:**
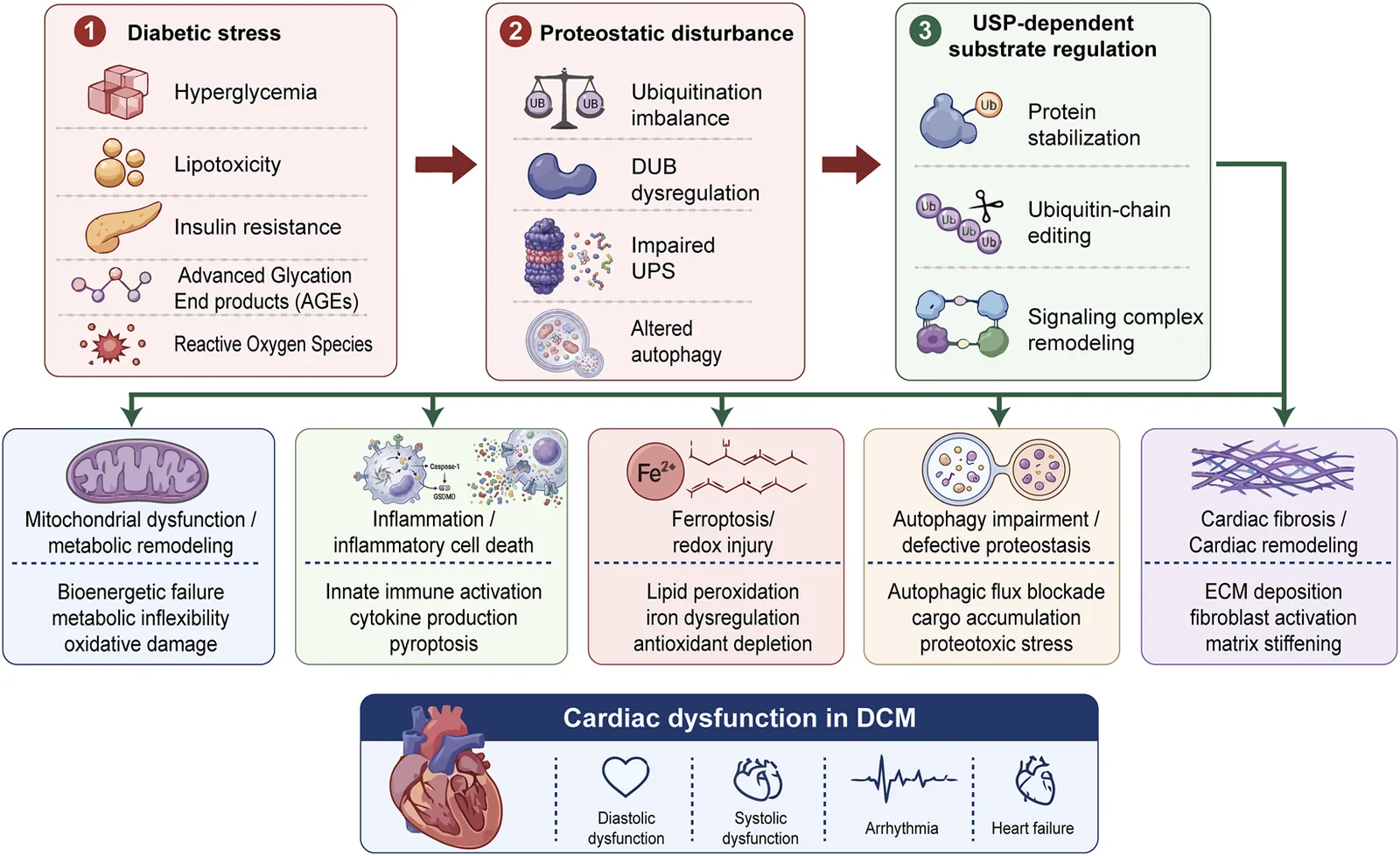
Conceptual framework: diabetic stress, proteostatic disturbance and USP-dependent remodeling in DCM. Diabetic stressors, including hyperglycaemia, lipotoxicity, insulin resistance, advanced glycation end-products (AGEs) and reactive oxygen species (ROS), disturb myocardial protein homeostasis through ubiquitination imbalance, deubiquitinase (DUB) dysregulation, impaired ubiquitin-proteasome system (UPS) activity and altered autophagy. USPs act on this disturbed proteostatic landscape through three mechanistic modes: protein stabilization, ubiquitin-chain editing, and signaling-complex remodeling. USP-dependent substrate regulation in turn links proteostatic failure to five interconnected pathological modules, mitochondrial dysfunction and metabolic remodeling, inflammation and inflammatory cell death, ferroptosis and redox injury, autophagy impairment and defective proteostasis, and fibrotic remodeling, which converge on diastolic dysfunction, systolic dysfunction, arrhythmia and heart failure. This framework reframes DCM as a disorder of metabolic stress-driven proteostatic maladaptation in which USPs function as nodal regulators rather than peripheral modifiers.

### 1.4 Literature search strategy

A focused narrative literature search was conducted in PubMed/MEDLINE, Web of Science Core Collection and Scopus from database inception to August 20th, 2026. To maximize sensitivity, the search did not rely solely on the terms “diabetic cardiomyopathy” and “USP”. Disease-related terms included “diabetic cardiomyopathy”, “diabetic heart”, “diabetic myocardial injury”, “diabetes-induced cardiac injury”, “db/db”, “HFD/STZ”, “high glucose” and “high glucose/free fatty acid”. These terms were combined with “ubiquitin-specific protease”, “ubiquitin-specific peptidase”, “deubiquitinase” and individual USP family-member symbols. Additional searches combined individual USPs with “cardiomyocyte”, “endothelial”, “mitochondria”, “mitophagy”, “ferroptosis”, “pyroptosis”, “autophagy”, “fibrosis” and “myocardial infarction”. Reference lists of relevant primary studies and reviews were manually screened. Evidence from non-ischemic DCM was distinguished from diabetic myocardial infarction, other cardiac injury models and non-cardiac mechanistic studies.

## 2 The ubiquitin-proteasome system, the ubiquitin code, and USP biology in cardiac proteostasis

Before examining USP-substrate axes in DCM, three foundational layers warrant brief consideration: the ubiquitin-proteasome system as the central machinery for cardiac protein quality control, the ubiquitin code as the language through which ubiquitin signals are interpreted, and USPs as active editors rather than passive antagonists of this code.

Cardiomyocytes are long-lived, highly energy-demanding cells that rely on efficient protein quality control to maintain sarcomeric organization, mitochondrial function, calcium handling and stress adaptation (Li et al., 2017; Nahum-Ankonina et al., 2023). The ubiquitin-proteasome system (UPS) is central to the selective turnover of damaged, misfolded, oxidized or short-lived regulatory proteins. Impaired UPS function has been directly linked to proteotoxic stress, maladaptive remodeling and cardiomyopathy, supporting the concept that disruption of cardiac proteostasis is an active contributor to myocardial disease rather than a secondary consequence (Su and Wang, 2010; Wang et al., 2011; Swatek and Komander, 2016; Pohl and Dikic, 2019). Ubiquitination is mediated by a hierarchical E1-E2-E3 enzymatic cascade, in which E3 ligases provide most of the substrate specificity.

Crucially, ubiquitination is not a single degradation tag but a combinatorial language. Different ubiquitin chain topologies encode functionally divergent outputs that are directly relevant to DCM: K48-linked chains classically target substrates for proteasomal degradation, whereas K63-linked chains more commonly regulate signaling-complex assembly, trafficking and selective autophagy, although non-canonical effects on substrate stability also occur in the diabetic heart, DNA-damage responses and receptor trafficking; M1-linked (linear) chains assembled by LUBAC and K11/K27/K33 atypical chains further fine-tune NF-κB activation, ERAD-related quality control and selective autophagy (Komander and Rape, 2012; Suryadinata et al., 2014; Swatek and Komander, 2016; Clague et al., 2019; Basar et al., 2021; Lange et al., 2022). Importantly for DCM, the same substrate may be modified by mechanistically distinct chains in different cellular contexts: NLRP3 is restrained by K48 chains in immune cells but activated by K63 chains in cardiomyocytes, and these distinct chain types are differentially edited by USP13 in different cellular contexts (Section 3.3); VDAC1 is targeted by non-canonical K63-mediated proteasomal degradation that USP14 reverses in diabetic cardiomyocytes (Section 4.4). This “ubiquitin code” enables cells to translate stress signals into rapid, reversible changes in protein stability, localization and complex assembly (Damgaard, 2021).

Deubiquitinating enzymes (DUBs) counterbalance ubiquitination by removing, trimming or editing ubiquitin chains. DUBs are not passive antagonists of ubiquitination but active regulators of ubiquitin-dependent signaling: by determining whether a substrate is degraded, stabilized, relocalized or incorporated into a signaling complex, DUBs shape the amplitude and duration of stress responses (Mevissen and Komander, 2017; Harrigan et al., 2018). Among DUBs, ubiquitin-specific proteases (USPs) form the largest family, with conserved cysteine protease catalytic domains and variable regulatory regions that determine substrate recognition, subcellular localization and chain-linkage preference (Suresh et al., 2020). Their context-dependent function in DCM is developed in the modular framework that follows. The reported USP-substrate axes and their evidence status are summarized in Table 1.

**TABLE 1:** Reported USP-substrate and downstream axes in diabetic cardiomyopathy and related diabetic myocardial injury.

USP-substrate/downstream axis	Functional interpretation	Ubiquitin linkage	Experimental model/context	Key evidence/limitation
USP28-PPARα → Mfn2	Protective axis	K48 on PPARα - demonstrated	db/db and HFD/STZ diabetic mice; cardiomyocyte-targeted AAV9-USP28; human diabetic myocardial samples	USP28 deubiquitinates and stabilizes PPARα at Lys152, sustaining Mfn2-dependent mitochondrial integrity and improving diabetic cardiac dysfunction. USP28 is downregulated in diabetic hearts (Xie et al., 2024)
USP13-NLRP3	Protective axis; cell type-dependent	K63 on NLRP3 - demonstrated	STZ and db/db mice; cardiomyocyte-specific knockout and AAV9-cTnT-USP13 rescue	USP13 removes activating K63-linked ubiquitin chains from NLRP3 at K557 and suppresses inflammasome assembly and pyroptosis in cardiomyocytes. Opposite NLRP3-stabilizing effects have been reported in macrophages outside DCM (Xu et al., 2026a; Li et al., 2025)
USP20-p62 → STING clearance	Protective axis	K63 on p62 - demonstrated	db/db and HFD/STZ mice; cardiomyocyte-specific USP20 loss-of-function; HG/PA-treated cardiomyocytes	USP20, through its catalytic C154 residue, removes K63-linked ubiquitination from p62 and facilitates p62-dependent autophagic clearance of STING (Zhou et al., 2026)
USP18-Notch1 → AKT	Protective axis	Unknown	STZ-induced diabetic rats; HG-treated AC16 cells	USP18 interacts with and stabilizes Notch1, with downstream AKT activation and reduced oxidative, inflammatory and mitochondrial injury. Ubiquitin-chain topology remains undefined (Cheng et al., 2025)
USP18-FOXC2 → JAK/STAT	Protective axis	Unknown	HG-treated neonatal mouse ventricular myocytes (*in vitro* only)	USP18 reduces FOXC2 ubiquitination and stabilizes FOXC2, suppressing pathological JAK2/STAT3 activation. Evidence is currently limited to an *in vitro* HG model, and chain topology remains undefined (Xu et al., 2026b)
USP7-PGC1β	Injurious axis (USP7 context-dependent)	Unknown	Diabetic mice; HG/PA-treated cardiomyocytes	USP7 interacts with and stabilizes PGC1β, promoting maladaptive metabolic remodeling and mitochondrial dysfunction. The relevant ubiquitin-chain topology was not defined (Yan et al., 2024)
USP7-NRF2 → GPX4	Protective axis (USP7 context-dependent; empagliflozin-associated)	K48 on NRF2 - demonstrated	Diabetic cardiomyopathy mice and cardiomyocytes during empagliflozin treatment	USP7 directly interacts with and deubiquitinates NRF2, reduces K48-linked ubiquitination, stabilizes NRF2 and supports GPX4-dependent ferroptosis resistance. USP7 inhibition attenuates empagliflozin-mediated protection (Cui et al., 2026)
USP8-Parkin	Protective, treatment-dependent axis	Unknown in the DCM study	db/db hearts; HG/oleate/palmitate-treated cardiomyocytes; H_2_S intervention	H_2_S-induced USP8 sulfhydration enhances USP8-Parkin interaction, Parkin deubiquitination, mitochondrial recruitment and mitophagy. The ubiquitin linkage was not defined in the DCM study (Sun et al., 2020)
USP33-ATG7 → FIS1	Protective, endothelial-specific axis	K63 on ATG7 - demonstrated	HG/FFA-treated human cardiac endothelial cells; endothelial-specific USP33-deficient HFD/STZ mice	USP33 limits K63-linked ubiquitination and stabilizes ATG7, enabling ATG7-dependent autophagic degradation of FIS1 and limiting mitochondrial fission and cardiac microvascular injury (Chen et al., 2026)
USP38-ACAD11 → RAGE	Injurious axis	K63 on ACAD11 - demonstrated	STZ diabetic mice with cardiomyocyte-specific USP38 gain/loss of function; HG-treated neonatal rat cardiomyocytes	USP38 directly interacts with ACAD11 and removes K63-linked ubiquitination, stabilizing ACAD11 and promoting fatty-acid metabolic/redox injury with downstream RAGE activation. The cognate E3 ligase remains unresolved (Xiao et al., 2025)
USP14-VDAC1	Injurious axis	K63 on VDAC1 - demonstrated	HG-treated AC16 cells; STZ-induced diabetic mice	USP14 directly deubiquitinates and stabilizes VDAC1, whereas ELAVL1 independently stabilizes VDAC1 mRNA. K63-linked editing is directly demonstrated; broader USP14 effects remain substrate- and disease-context dependent (Wang et al., 2026)
USP24-associated NF-κB signaling	Injurious, pathway-level evidence	Unknown	HFD/STZ mice; HG/PA-treated cardiomyocytes	USP24 acts functionally upstream of NF-κB and promotes ferroptotic injury. The direct USP24 substrate and the relevant ubiquitin linkage remain unresolved (Wu et al., 2024)
USP24-TRAF6 → Beclin-1	Injurious axis	Unknown for USP24→TRAF6	HFD/STZ mice; primary cardiomyocytes and H9c2 cells	USP24 directly deubiquitinates and stabilizes TRAF6; TRAF6 subsequently ubiquitinates Beclin-1 and promotes its degradation, impairing autophagy. The ubiquitin linkage removed from TRAF6 by USP24 remains undefined (Wu et al., 2026)

“Demonstrated” indicates that the stated deubiquitination event or ubiquitin linkage was directly tested in the cited study. “Unknown” indicates that the corresponding ubiquitin linkage was not defined. “Pathway-level” indicates that functional regulation of the pathway was demonstrated but the direct USP, substrate remains unresolved. The symbol “→” denotes a downstream effector or signaling consequence rather than a directly deubiquitinated substrate.

## 3 USP-substrate axes with predominantly protective effects in DCM

### 3.1 How protective USP-substrate axes buffer diabetic myocardial stress

In this section, “protective” refers to the observed effect of a defined USP-substrate axis in the experimental context studied and not to an invariant property of the USP enzyme. Current evidence identifies several such axes that preserve mitochondrial quality, restrain inflammatory cell death, promote autophagic or mitophagic clearance, or maintain stress-adaptive signaling.

These protective mechanisms are mechanistically diverse. USP28 stabilizes PPARα to preserve Mfn2-dependent mitochondrial integrity (Xie et al., 2024); USP8 facilitates Parkin-dependent mitophagy in the diabetic heart (Sun et al., 2020); USP13 edits NLRP3 ubiquitination to restrain cardiomyocyte pyroptosis (Xu D. et al., 2026); USP20 licenses selective autophagic clearance of STING (Zhou et al., 2026), USP18 stabilizes Notch1 or FOXC2 in diabetic cardiomyocyte injury (Cheng et al., 2025; Xu Z. et al., 2026), and endothelial USP33 stabilizes ATG7 to limit FIS1-dependent mitochondrial fission (Chen et al., 2026). Thus, the shared feature is not a common chain preference or mode of deubiquitination, but the preservation of an adaptive substrate program in a defined cardiac compartment.

This axis-level definition is essential because the same enzyme can produce different effects through different substrates or cell types. Protective classification should therefore always be interpreted together with substrate identity, cardiac cell type and disease model. Sections 3.2–3.7 examine each protective USP under this lens, with emphasis on substrate identity, ubiquitin-chain topology and the cellular and disease-stage contexts within which protection is operative.

### 3.2 USP28: mitochondrial morphofunctional integrity via PPARα-Mfn2

USP28 is currently the most mechanistically defined protective USP in DCM. Using db/db and HFD/STZ-induced diabetic models with cardiomyocyte-specific AAV9-USP28 delivery, Xie and colleagues demonstrated that USP28 preserves mitochondrial morphofunctional integrity and improves systolic and diastolic dysfunction in the diabetic heart (Xie et al., 2024). Mechanistically, USP28 deubiquitinates and stabilizes PPARα at lysine 152 by removing K48-linked polyubiquitin chains, thereby sustaining PPARα-dependent transcriptional activation of Mfn2 and preserving the mitochondrial fusion-fission balance that diabetic stress otherwise disrupts. Critically, USP28 expression is significantly downregulated in db/db mouse hearts and in human diabetic myocardial samples (Xie et al., 2024), which is consistent with loss of this protective USP28-PPARα/Mfn2 axis under diabetic stress.

The conceptual significance of the USP28-PPARα-Mfn2 axis extends beyond mitochondrial dynamics alone. PPARα is itself a Janus-faced regulator in the diabetic heart: when coupled to efficient mitochondrial function and balanced substrate utilization, PPARα-driven fatty acid oxidation supports energy homeostasis; when uncoupled or excessive, it reinforces lipid overload, ROS generation and electron-transport-chain pressure (Finck et al., 2002; Lee et al., 2017). The same logic applies at the post-translational layer. PPARα stabilization is not intrinsically protective, as demonstrated by the maladaptive USP7-PGC1β axis discussed in Section 4.2, where stabilization of a PPAR-family coactivator drives injury rather than protection. What distinguishes USP28 is that PPARα stabilization is functionally coupled to Mfn2 induction, meaning that the metabolic program licensed by USP28 includes the mitochondrial structural capacity to handle increased oxidative flux. This functional coupling exemplifies the protective axis framework articulated in Section 3.1: protective USPs stabilize substrates whose downstream effectors perform adaptive work, in this case mitochondrial fusion and quality control (Suliman and Piantadosi, 2016).

### 3.3 USP13: cardiomyocyte protection through cell type-restricted NLRP3 deubiquitination

USP13 provides the most mechanistically informative example of cell type-dependent USP biology in DCM. Using cardiomyocyte-specific Usp13 knockout and AAV9-cTnT-USP13 rescue in both type I (STZ) and type II (db/db) diabetic mouse models, Xu and colleagues demonstrated that USP13 is predominantly expressed in cardiomyocytes within the diabetic heart, is significantly downregulated by diabetic stress, and, when restored, deubiquitinates NLRP3 to suppress caspase-1-dependent gasdermin D cleavage and limit pyroptotic cardiomyocyte death (Xu D. et al., 2026). This downregulation pattern conforms to the protective axis framework: loss rather than gain of deubiquitination drives disease.

The broader cardiomyocyte-protective role of USP13 extends beyond inflammasome regulation. In pressure overload-induced hypertrophy, USP13 deubiquitinates and stabilizes STAT1 to preserve mitochondrial function and restrain hypertrophic remodeling (Han et al., 2025). The convergence of two independent substrate axes, NLRP3 in DCM, STAT1 in hypertrophy, on cardiomyocyte protection suggests that USP13 functions as a multi-substrate resilience factor, with the operative USP-substrate pairing selected by the dominant stress context rather than by enzymatic preference alone.

The conceptually most important feature of USP13 biology is its opposing function in immune cells, and resolving this paradox at the ubiquitin-chain level informs every aspect of subsequent therapeutic design. In macrophages, the E3 ligase TRIM31 catalyzes K48-linked polyubiquitination of NLRP3 to target it for proteasomal degradation (Song et al., 2016). USP13 antagonizes this degradative signal by cleaving the K48 chain, stabilizing NLRP3 and facilitating inflammasome activation (Li et al., 2025). In diabetic cardiomyocytes, by contrast, NLRP3 priming involves non-degradative K63-linked ubiquitination, and USP13-mediated removal of these activating K63 chains disassembles inflammasome-competent NLRP3 complexes without affecting NLRP3 protein abundance (Xu D. et al., 2026). The same enzyme acts on the same substrate but edits different ubiquitin-chain types assembled by different E3 ligases in different cell types, producing opposite biological outcomes. This is currently the clearest mechanistic illustration of context-dependent USP biology in cardiovascular disease, generalizing the principle first established for BRCC3-mediated NLRP3 deubiquitination (Py et al., 2013): NLRP3 inflammasome activity is governed by chain-linkage-specific deubiquitination rather than by total substrate abundance alone.

This cell type- and chain-resolved duality has important translational implications. Systemic USP13 modulation would produce opposing effects in cardiomyocytes and in tissue-resident or infiltrating macrophages, with unpredictable net outcomes depending on which compartment dominates the diabetic myocardial inflammatory milieu. These opposing cell type-specific effects indicate that any future attempt to modulate USP13 will require cardiomyocyte-versus-immune-cell target engagement to be resolved experimentally before systemic intervention can be considered.

### 3.4 USP20: STING clearance and selective autophagy licensing

USP20 occupies a distinctive position among protective USP-substrate axes because its cardioprotective function operates not by stabilizing a protective effector, but by licensing the selective autophagic clearance of an inflammatory signaling complex. This places USP20 within the signaling-complex remodeling mode of protective USP biology, alongside USP13 and distinct from the substrate-abundance mode of USP28.

The foundational autophagy-supportive activities of USP20 were established outside DCM. USP20 deubiquitinates and stabilizes ULK1 to support autophagy initiation (Kim et al., 2018), and drives selective endoplasmic reticulum autophagy by cleaving K48- and K63-linked ubiquitin chains on the ER-phagy receptor RETREG1/FAM134B (Zhang M. et al., 2024). These findings establish USP20 as a multi-layered autophagy-licensing enzyme acting both at upstream initiation and at selective cargo recognition.

In DCM, Zhou and colleagues demonstrated that cardiomyocyte-derived USP20 directly binds STING and promotes its autophagy-dependent degradation by deubiquitinating p62 through the catalytic C154 residue of USP20 (Zhou et al., 2026). The mechanistic feature critical for therapeutic conceptualization is that USP20 does not block STING activation; rather, it ensures that activated STING is cleared after signaling, preventing the persistent type-I interferon and NF-κB amplification that drives diabetic myocardial sterile inflammation. USP20 thus acts as a proteostatic termination mechanism for innate immune signaling, suppressing pathway persistence rather than pathway output, a conceptually distinct strategy from conventional anti-inflammatory pharmacology.

USP20’s cardioprotective relevance extends beyond DCM. Cardiomyocyte-enriched USP20 also attenuates pressure overload-induced hypertrophy through STAT3 deubiquitination, providing cross-cardiac support for its stress-adaptive function (Zhong et al., 2025). In vascular smooth muscle cells, USP20 attenuates TNF- and IL-1β-induced atherogenic signaling through RIPK1 deubiquitination (Jean-Charles et al., 2018), directionally aligned with the cardiomyocyte anti-inflammatory phenotype. The directionally consistent effects observed in cardiomyocytes and vascular smooth muscle cells are notable; however, USP20 function in cardiac fibroblasts, endothelial cells and infiltrating macrophages remains undefined, precluding confident prediction of systemic effects.

### 3.5 USP18: convergent protection through Notch1-AKT and FOXC2-JAK/STAT signaling

Current evidence supports two protective USP18-dependent pathways in diabetic myocardial injury. Cheng and colleagues demonstrated that USP18 restoration in high glucose (HG)-treated AC16 cells and diabetic rat hearts deubiquitinates and stabilizes Notch1, activates downstream AKT signaling, and reduces oxidative stress, inflammation, mitochondrial impairment and myocardial injury (Cheng et al., 2025). A second study using HG-treated neonatal mouse ventricular myocytes similarly found that USP18 was downregulated by diabetic stress and that USP18 overexpression increased cell viability while reducing apoptosis, inflammatory responses, oxidative stress and fibrosis-related changes. Mechanistically, USP18 reduced FOXC2 ubiquitination and stabilized FOXC2. FOXC2 knockdown reversed these protective effects and restored HG-induced JAK2/STAT3 activation (Xu Z. et al., 2026). The available evidence suggests that USP18 can protect diabetic cardiomyocytes through at least two distinct substrate pathways. Whether Notch1 and FOXC2 are engaged simultaneously or under different metabolic conditions remains unresolved.

### 3.6 USP8: H_2_S-dependent sulfhydration links deubiquitination to Parkin-mediated mitophagy

USP8 represents an earlier link between deubiquitination and mitochondrial quality control in the diabetic heart. In db/db mice, USP8 expression and S-sulfhydration were reduced, accompanied by increased cytosolic Parkin ubiquitination and impaired mitochondrial recruitment of Parkin (Sun et al., 2020). Exogenous H_2_S restored USP8 S-sulfhydration, enhanced USP8-Parkin interaction and Parkin deubiquitination, promoted mitochondrial Parkin translocation and mitophagy, and improved mitochondrial and cardiac function (Sun et al., 2020).

This study is mechanistically notable because it introduces regulation of the DUB itself by another post-translational modification. However, the protective mechanism was demonstrated primarily in the context of H_2_S treatment rather than through cardiomyocyte-specific genetic restoration of USP8. USP8-Parkin should therefore be considered a treatment-dependent protective mitophagy axis with direct relevance to diabetic hearts but less genetic validation than the USP28, USP13 or USP20 pathways.

### 3.7 USP33: endothelial ATG7-FIS1 control of mitochondrial fission and microvascular injury

Recent evidence extends DCM-related USP biology beyond cardiomyocytes. Chen and colleagues identified USP33 as a regulator of cardiac microvascular endothelial mitochondrial homeostasis using high glucose/free fatty acid-treated human cardiac endothelial cells and endothelial-specific USP33-deficient mice subjected to HFD/STZ-induced diabetes (Chen et al., 2026). Mechanistically, USP33 stabilizes ATG7 by limiting K63-linked ubiquitination, thereby promoting ATG7-dependent autophagic degradation of FIS1. Loss of USP33 increases FIS1 abundance, excessive mitochondrial fission, mitochondrial dysfunction and endothelial injury. In diabetic mice, endothelial USP33 deficiency further impairs microvascular density, perfusion and endothelial signaling and aggravates cardiac dysfunction and remodeling (Chen et al., 2026).

The USP33-ATG7/FIS1 axis therefore provides direct evidence that USP-dependent proteostatic regulation in DCM is not restricted to cardiomyocytes. It establishes cardiac microvascular endothelium as a second major cellular compartment in which USP activity can influence the progression of diabetic myocardial disease.

## 4 USP-substrate axes with predominantly injurious or bidirectional effects in DCM

### 4.1 Overview

Current evidence identifies USP38-ACAD11 (Xiao et al., 2025), USP14-VDAC1 (Wang et al., 2026) and USP24-associated signaling (Wu et al., 2024; Wu et al., 2026) as axes that predominantly amplify diabetic myocardial injury in the experimental contexts studied. USP7 requires separate consideration because its biological effect differs according to substrate identity. The USP7-PGC1β axis promotes maladaptive metabolic remodeling and mitochondrial dysfunction (Yan et al., 2024), whereas USP7-mediated stabilization of NRF2 supports antioxidant and anti-ferroptotic protection during empagliflozin treatment (Cui et al., 2026).

Accordingly, this section classifies the USP-substrate axis, rather than the USP enzyme, as predominantly injurious, protective or bidirectional. This distinction is particularly important for therapeutic interpretation because whole-enzyme inhibition may suppress an injurious substrate pathway while simultaneously disrupting a protective one.

### 4.2 USP7: substrate-dependent bidirectionality between PGC1β and NRF2

USP7 provides the clearest example of substrate-dependent bidirectionality in DCM. Yan and colleagues demonstrated that USP7 is significantly upregulated in diabetic mouse hearts and HG/palmitate-treated cardiomyocytes, and USP7 interacts with and stabilizes PGC1β, thereby sustaining PPARα-associated fatty acid metabolic programs, mitochondrial fission and impaired mitochondrial homeostasis. Genetic or pharmacological suppression of USP7 improved these phenotypes, supporting an injurious USP7-PGC1β axis (Yan et al., 2024). The stabilized PGC1β sustains mitochondrial oxidative metabolism and fatty acid utilization programs that, in a lipid-overloaded diabetic heart, drive excessive fatty acid oxidation, increased mitochondrial workload and oxygen consumption, and amplified ROS generation, ultimately aggravating cardiometabolic dysfunction and mitochondrial homeostasis impairment (Haemmerle et al., 2011). In contrast, Cui and colleagues identified a protective USP7-NRF2 axis during empagliflozin treatment (Cui et al., 2026). Empagliflozin increased NRF2 abundance, GPX4 expression and resistance to cardiomyocyte ferroptosis. USP7 interacted with NRF2 and mediated its deubiquitination, thereby stabilizing NRF2. NRF2 deficiency abolished the cardiac and anti-ferroptotic effects of empagliflozin, whereas pharmacological inhibition of USP7 with P5091 attenuated these benefits and AAV9-mediated NRF2 restoration rescued the phenotype (Cui et al., 2026).

These studies demonstrate that USP7 cannot be classified as uniformly maladaptive. Its net effect depends on whether PGC1β-driven metabolic reprogramming or NRF2-dependent antioxidant defense is the dominant substrate program. Whole-enzyme USP7 inhibition could therefore suppress an injurious metabolic axis while simultaneously weakening NRF2/GPX4-mediated ferroptosis resistance. Future studies should determine how diabetic stress and cardiometabolic treatment alter USP7 substrate engagement and whether the PGC1β and NRF2 programs compete for USP7 activity.

The conceptual significance is best understood by direct comparison with USP28. Both DUBs regulate components of the PPAR/PGC-1 metabolic transcriptional network, but through distinct substrates (Section 3.2): USP28 stabilizes PPARα, whereas USP7 stabilizes PGC1β. The mechanistic difference lies in functional coupling: USP28-mediated PPARα stabilization is coupled to Mfn2 induction and mitochondrial fusion capacity, providing the structural machinery to handle increased oxidative flux; USP7-mediated PGC1β stabilization activates oxidative metabolism without compensatory mitochondrial structural support, producing flux-capacity mismatch. This contrast reinforces a broader principle articulated throughout this review: components of the same metabolic transcriptional network can be either protective or pathogenic depending on whether their stabilization is coupled to or uncoupled from supporting structural and metabolic infrastructure (Finck et al., 2002; Yin et al., 2019). These findings make USP7 a useful model for understanding substrate-selective DUB regulation, while arguing against indiscriminate whole-enzyme inhibition in DCM.

### 4.3 USP38: ACAD11-dependent fatty acid oxidation and redox-metabolic injury

USP38 extends the injurious axis framework from transcriptional coactivation to direct metabolic enzyme regulation. Xiao and colleagues demonstrated that USP38 is upregulated in diabetic hearts and exacerbates DCM through post-translational stabilization of ACAD11, a long-chain fatty acid oxidation enzyme (Xiao et al., 2025). The stabilized ACAD11 amplifies fatty acid oxidation flux beyond mitochondrial respiratory capacity, increasing electron transport chain pressure, ROS production and lipid peroxidation (Paquay et al., 2024), a redox-metabolic injury profile mechanistically distinct from the broader transcriptional reprogramming driven by USP7-PGC1β.

The USP38-ACAD11 axis demonstrates that maladaptive deubiquitination operates not only at the transcriptional layer (USP7) but also at the metabolic enzyme layer, expanding the substrate repertoire of injurious DCM-relevant USPs. Notably, USP38 has also been linked to diabetes-related atrial fibrillation through stabilization of iron regulatory protein 2, raising electrical remodeling as an additional pathological dimension (Xiao et al., 2024). The convergence of structural cardiac remodeling in DCM and electrical remodeling in atrial fibrillation suggests that USP38 may participate in multiple injurious diabetic cardiac programs, although the relevant substrates differ across contexts (ACAD11 vs. IRP2) (Xiao et al., 2024; Xiao et al., 2025).

Two caveats warrant emphasis. First, the DCM-relevant USP38-ACAD11 axis currently rests on a single mechanistic study, and independent replication in db/db or HFD/STZ models is required before this axis can be incorporated into therapeutic decision-making. Second, the cited study directly demonstrated that USP38 reduces K63-linked polyubiquitination of ACAD11, establishing K63 as the experimentally supported linkage in this axis; however, the cognate E3 ligase remains unresolved.

### 4.4 USP14: dual-layer stabilization of VDAC1 with ELAVL1 as a multi-modular amplifier

USP14 represents the most recently characterized maladaptive USP in DCM and introduces a regulatory architecture previously absent from the DCM-USP literature: cooperative dual-layer stabilization between a deubiquitinase and an RNA-binding protein. Wang and colleagues demonstrated that USP14 and ELAVL1 (HuR) are both upregulated in HG-treated AC16 cardiomyocytes and STZ-induced diabetic mice, and that they converge on the mitochondrial outer-membrane channel VDAC1 through complementary mechanisms: ELAVL1 binds the VDAC1 3′-UTR and stabilizes VDAC1 mRNA, while USP14 directly interacts with VDAC1 protein and removes ubiquitin chains to prevent proteasomal degradation (Wang et al., 2026).

The mechanistic detail most informative for the broader ubiquitin code is that USP14 removes K63-linked rather than K48-linked chains from VDAC1, yet the functional consequence is still proteasomal protection (MG132 rescues VDAC1 protein levels when USP14 is depleted) (Wang et al., 2026). This represents a non-canonical K63-mediated degradation pathway, an exception to the classical K48-degradative paradigm that nonetheless preserves the substrate-abundance-rescue functional principle of the injurious substrate-stabilization pattern. The downstream consequences of VDAC1 stabilization are notably multi-modular: increased VDAC1 simultaneously drives apoptosis (Bcl-2/Bax imbalance), inflammation (IL-6, IL-1β release), ferroptosis (Fe^2+^, lipid ROS, ACSL4 elevation with GPX4 suppression) and mitochondrial membrane potential collapse, all reversed by VDAC1 knockdown.

The cardiac role of USP14 is itself context-dependent and provides one of the cleanest examples of substrate competition. In doxorubicin-induced cardiotoxicity, USP14 deubiquitinates and stabilizes SIRT3 to preserve mitochondrial integrity and limit pyroptosis (Zhang Z. et al., 2024). The opposing effects of USP14 in DCM and doxorubicin cardiotoxicity further reinforce the central principle that USP function is determined by substrate identity and disease context rather than by enzyme activity alone. The identification of the USP14-VDAC1 axis provides a pharmacological hypothesis rather than an established therapeutic strategy. USP14-directed compounds such as IU1 and related analogues provide useful chemical tools for testing whether suppression of USP14 can destabilize VDAC1 and attenuate diabetic myocardial injury (Lee et al., 2010). By contrast, b-AP15 inhibits both USP14 and UCHL5 and induces broader proteotoxic stress and should therefore not be interpreted as a selective USP14 intervention (Zhang et al., 2018; Jiang et al., 2022). Whether selective USP14 modulation achieves sufficient myocardial exposure, preserves beneficial USP14 substrates and improves DCM without chronic proteostatic toxicity remains unknown.

### 4.5 USP24: NF-κB-associated ferroptosis and TRAF6-dependent autophagy suppression

USP24 has been implicated in two injurious processes in experimental DCM, but the underlying mechanisms are distinct. Wu and colleagues demonstrated that USP24 was upregulated in diabetic hearts and high glucose/palmitate-treated cardiomyocytes, and USP24 silencing reduced NF-κB activation and ferroptotic injury (Wu et al., 2024). These findings place USP24 functionally upstream of NF-κB; however, the direct substrate linking USP24 deubiquitination to NF-κB regulation was not identified. The same group subsequently identified a separate USP24-dependent mechanism in autophagy. USP24 directly interacted with and deubiquitinated TRAF6, thereby stabilizing TRAF6 protein. Stabilized TRAF6 subsequently promoted Beclin-1 ubiquitination and degradation, resulting in impaired autophagic flux. Silencing USP24 reduced TRAF6 abundance, restored Beclin-1 levels, enhanced autophagy and improved cardiomyocyte viability, whereas TRAF6 overexpression reversed these effects (Wu et al., 2026). Thus, TRAF6, not Beclin-1, is the directly demonstrated USP24 substrate in this pathway. The ubiquitin-chain linkage removed from TRAF6 by USP24 was not fully defined in the cited DCM study and should therefore be considered unresolved.

A context-dependence caveat applies: in drug-resistant cancer cell models, USP24 inhibition induces ferroptosis rather than suppressing it (Wang et al., 2024), reflecting tissue- and disease-context-specific substrate engagement. Selective USP24 inhibitors remain at an early development stage.

## 5 Underexplored but mechanistically plausible candidates

### 5.1 USP30 and mitophagy in the diabetic heart

An important gap in the current DCM-USP literature concerns USP30, a mitochondrial outer-membrane-anchored deubiquitinase that opposes Parkin- and PINK1-driven mitophagy by removing K6- and K11-linked ubiquitin chains from damaged mitochondrial proteins (Bingol et al., 2014). Mitophagy failure is increasingly recognized as a core mechanism of diabetic cardiac mitochondrial dysfunction, with impaired clearance of damaged mitochondria amplifying ROS generation, lipid peroxidation and bioenergetic decline (Cai et al., 2022). Pharmacological USP30 inhibition has been shown to enhance cardiac mitophagy in non-diabetic mice without overt impairment of cardiac function, providing proof that systemic USP30 modulation can influence mitochondrial quality control in the heart (Luo et al., 2021). However, these studies did not evaluate DCM, and quantitative myocardial exposure, target engagement and therapeutic efficacy in diabetic myocardium remain undefined. USP30 should therefore be regarded as a mechanistically plausible candidate for future DCM investigation rather than an immediately repurposable therapeutic target.

### 5.2 Proteasome-associated DUBs and cardiac proteinopathy: brief outlook

The maladaptive role of USP14 in DCM (Section 4.4) raises a broader question of whether other proteasome-associated deubiquitinases similarly contribute to diabetic cardiac proteinopathy. The diabetic heart accumulates oxidized, glycated and misfolded proteins that progressively overload proteasomal capacity, and the proteasome-anchored DUBs USP14, UCH37 and POH1 together fine-tune substrate flux through the 26S proteasome (Pohl and Dikic, 2019). Whether UCH37 or POH1 contributes to DCM through mechanisms distinct from the USP14-VDAC1 axis is currently unknown but represents a mechanistically defined direction for systematic investigation, particularly given that proteasome-targeted small molecules already exist as candidate cardiac proteostasis modulators.

### 5.3 Ubiquitin-like modifications adjacent to USP biology in DCM

Although this review focuses on USP-dependent deubiquitination, diabetic myocardial proteostasis is also shaped by ubiquitin-like post-translational modifications, including UFMylation, neddylation and SUMOylation. These systems regulate ER homeostasis, cullin-RING ligase activity, calcium handling, mitochondrial stress and autophagic quality control; therefore, they should be viewed as adjacent proteostatic layers that may intersect with, rather than replace, USP-regulated modules in DCM (Li et al., 2018; Li et al., 2020; Millrine et al., 2023). UFMylation is closely linked to ER proteostasis, ribosome-associated quality control, ER-phagy and the unfolded protein response (Millrine et al., 2023). In the heart, the UFM1-specific ligase UFL1 is required for maintaining ER homeostasis and cardiac function; UFL1 deficiency provokes ER stress, cardiomyopathy, fibrosis and contractile dysfunction (Li et al., 2018). Although direct evidence linking UFMylation to DCM remains limited, this pathway is mechanistically relevant because ER stress and defective proteostasis are core features of diabetic myocardial injury.

Neddylation regulates cullin-RING ligases and thereby shapes the ubiquitination landscape on which USPs act. Cardiac neddylation has been implicated in cardiac development, function and protein quality control (Li et al., 2020), and inhibition of the NEDD8-activating enzyme by MLN4924 attenuates oxidative injury, fibrosis and mitochondrial dysfunction in doxorubicin-induced cardiotoxicity (Chen et al., 2024). DCM-specific neddylation studies remain scarce, but altered neddylation could theoretically modify USP biology by changing E3 ligase-dependent substrate availability.

SUMOylation has more direct relevance to diabetic myocardial dysfunction. Hydrogen sulfide has been reported to improve systolic-diastolic function in DCM by regulating SERCA2a SUMOylation through S-sulfhydration of SENP1 (Peng et al., 2023). In addition, UBC9/SUMOylation supports cardiac protein quality control in stressed hearts (Gupta et al., 2016), and recent DCM-related work suggests that UBC9 ameliorates defective cardiomyocyte mitophagy through the NEDD4/RUNX2/PSEN2 axis (Wu et al., 2025). Thus, SUMOylation may intersect with USP biology through calcium cycling, mitophagy and proteostatic adaptation.

Together, these ubiquitin-like modifications broaden the proteostatic framework of DCM. They underscore that diabetic myocardial remodeling is governed by a larger post-translational network in which ubiquitination, deubiquitination, UFMylation, neddylation and SUMOylation converge on ER stress, mitochondrial quality control, autophagy and regulated cell death.

## 6 Mapping USP biology onto DCM pathological modules

Sections 3–5 examined USPs on an enzyme-by-enzyme basis. Here we invert the perspective: rather than asking what each USP does, we ask which USP-substrate axes converge on each DCM pathological program, and how protective and maladaptive axes interact, compete or amplify each other within a given module. This synthesis is important because DCM pathology is organized at the level of interacting pathological programs rather than isolated enzymatic activities. USPs detailed above are referenced by their substrate axis without restating mechanism. The integration of protective and maladaptive USP axes across these pathological modules is depicted in Figure 2.

**FIGURE 2:**
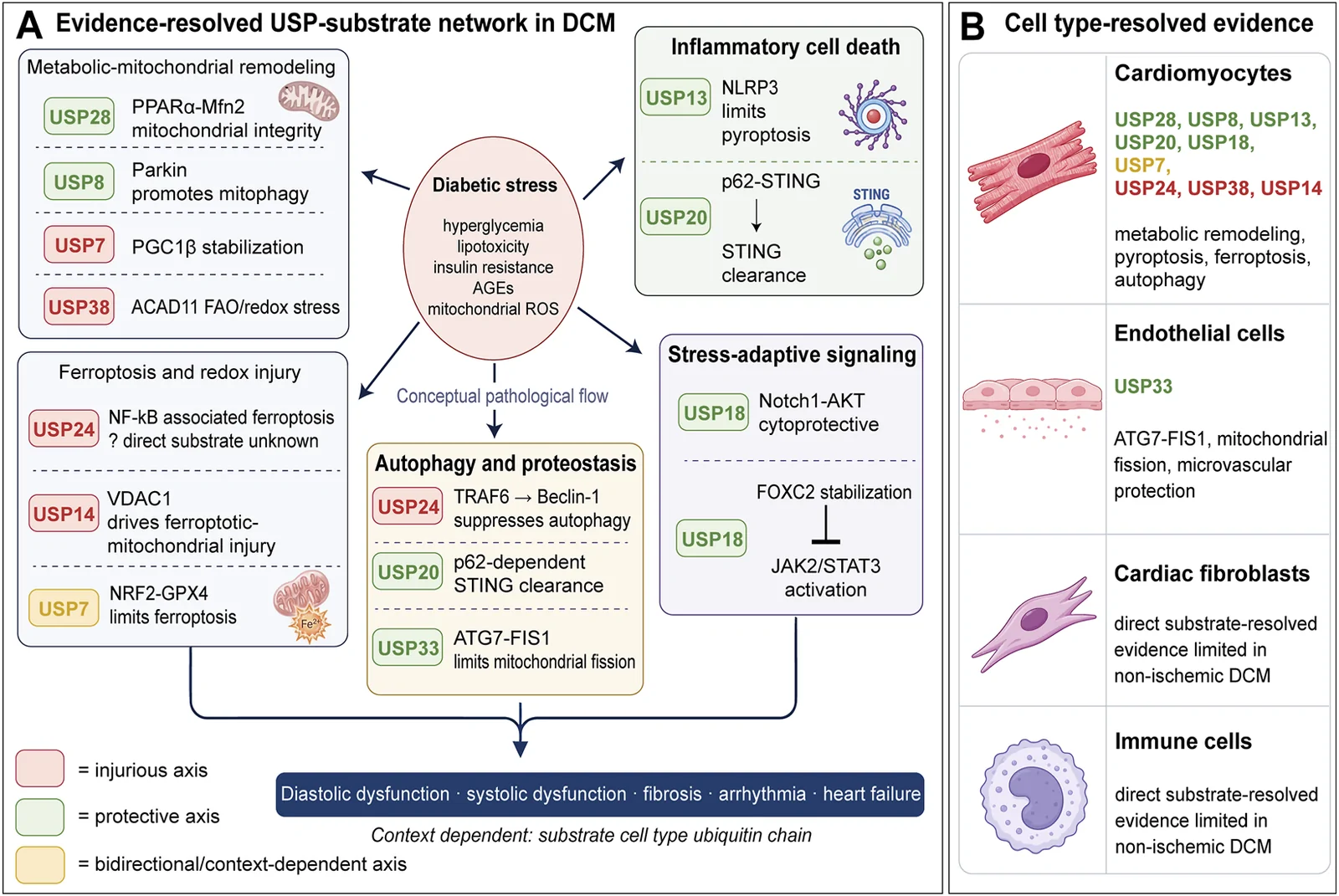
Evidence-resolved USP-substrate network in diabetic cardiomyopathy. **(A)** Experimentally supported USP-substrate axes are mapped onto major pathological processes in DCM. Green denotes predominantly protective axes, red denotes predominantly injurious axes, and amber denotes bidirectional or context-dependent regulation. Question marks identify unresolved direct substrates or ubiquitin-linkage information. USP7 illustrates substrate-dependent bidirectionality through the injurious USP7-PGC1β axis and the protective USP7-NRF2/GPX4 axis. The USP24 autophagy pathway is represented as direct stabilization of TRAF6 followed by TRAF6-dependent Beclin-1 ubiquitination and degradation. **(B)** Cell type-resolved evidence. Most direct DCM evidence derives from cardiomyocytes, whereas USP33 provides direct endothelial-specific evidence through the ATG7-FIS1 pathway. Direct substrate-resolved USP evidence in cardiac fibroblasts and immune cells remains limited in non-ischemic DCM. The previous stage-specific panel was removed because temporal dominance of individual USP axes has not been directly established experimentally.

### 6.1 Mitochondrial dysfunction and metabolic remodeling

The mitochondrial module contains both cardiomyocyte- and endothelial-specific USP-substrate axes. USP28 preserves PPARα-Mfn2-dependent mitochondrial integrity (Xie et al., 2024), while the H_2_S-USP8-Parkin pathway promotes mitophagic clearance of damaged mitochondria (Sun et al., 2020). More recently, endothelial USP33 was shown to stabilize ATG7 and promote FIS1 autophagic degradation, thereby limiting excessive mitochondrial fission and cardiac microvascular dysfunction (Chen et al., 2026).

These protective pathways are opposed by several injurious substrate programs. USP7 stabilizes PGC1β to drive flux-capacity mismatch (Yan et al., 2024), USP38 stabilizes ACAD11 to amplify fatty acid oxidation-dependent redox injury (Xiao et al., 2025), and USP14 stabilizes VDAC1 to drive outer-membrane permeabilization (Wang et al., 2026). Importantly, USP7 itself is not uniformly injurious because its NRF2 substrate program contributes to ferroptosis resistance. The mitochondrial phenotype in DCM should therefore be interpreted as the net result of multiple substrate-specific proteostatic programs rather than a simple balance between protective and maladaptive enzymes.

### 6.2 Inflammatory cell death: NLRP3 and STING signaling

The inflammatory module in DCM is organized around two innate immune signaling axes, NLRP3 inflammasome activation and cGAS-STING-driven type-I interferon signaling, each engaged by a distinct protective-maladaptive USP pair. On the NLRP3 axis, USP13 (Section 3.3) disassembles activating K63 chains on NLRP3 to restrain cardiomyocyte pyroptosis (Xu D. et al., 2026), while USP24 acts functionally upstream of NF-κB and amplifies NF-κB-associated inflammatory and ferroptotic signaling, although the direct USP24 substrate responsible for this regulation remains unresolved (Wu et al., 2024). On the STING axis, USP20 (Section 3.4) licenses selective autophagy that clears activated STING (Zhou et al., 2026). USP14, by contrast, contributes to inflammatory injury through VDAC1-dependent mitochondrial dysfunction, although a direct mechanistic connection between the USP14-VDAC1 axis and cGAS-STING signaling has not been established in DCM (Wang et al., 2026). The conceptual feature distinguishing this module from the mitochondrial module is that these USP-substrate axes act at different functional positions within inflammatory signaling. USP20 promotes signaling termination through STING clearance, whereas USP13 restrains inflammasome activation through K63 editing of NLRP3. In contrast, USP24 amplifies NF-κB-associated inflammatory injury, while USP14 contributes to inflammation through VDAC1-dependent mitochondrial dysfunction, although its direct relationship with cGAS-STING signaling remains unresolved in DCM.

### 6.3 Ferroptosis and redox injury

Ferroptosis in DCM is driven by two mechanistically distinct USP-dependent maladaptive axes that operate at different molecular layers. The USP24-NF-κB axis (Section 4.5) acts at the transcriptional-inflammatory layer, lowering the threshold for iron-dependent lipid peroxidation through sustained NF-κB activation (Wu et al., 2024). The USP14-ELAVL1-VDAC1 axis (Section 4.4) acts at the mitochondrial structural layer, stabilizing the outer-membrane channel whose oligomerization drives mitochondrial ROS overflow, GPX4 suppression and ACSL4 induction (Wang et al., 2026). In contrast, the USP7-NRF2/GPX4 axis (Section 4.2) provides direct evidence for a protective deubiquitination pathway. During empagliflozin treatment, USP7 stabilizes NRF2, supports GPX4 expression and limits cardiomyocyte ferroptosis. Pharmacological inhibition of USP7 attenuates this protective effect. The ferroptosis module therefore illustrates substrate-dependent bidirectionality rather than an exclusively maladaptive USP program.

### 6.4 Autophagy, mitophagy and proteostatic clearance

USP-dependent regulation of autophagy in DCM operates at several distinct levels. USP20 (Section 3.4) facilitates selective autophagic clearance of STING through regulation of p62, thereby limiting persistent inflammatory signaling (Zhou et al., 2026). In contrast, USP24 (Section 4.5) directly deubiquitinates and stabilizes TRAF6; accumulated TRAF6 subsequently promotes Beclin-1 ubiquitination and degradation, resulting in impaired autophagic flux (Shi and Kehrl, 2010; Wu et al., 2026). Beyond the USP20 and USP24 pathways, two additional USPs expand the autophagy-related DCM network. H_2_S-dependent sulfhydration of USP8 (Section 3.6) enhances Parkin deubiquitination and promotes mitophagy in db/db hearts, whereas endothelial USP33 (Section 3.7) stabilizes ATG7 and facilitates ATG7-dependent autophagic degradation of FIS1.

Together, these findings indicate that USP-dependent control of proteostatic clearance in DCM spans selective inflammatory cargo removal, mitophagy, autophagy regulation and endothelial mitochondrial quality control. The functional outcome is determined primarily by substrate identity and position within the proteostatic network rather than by a shared ubiquitin-chain preference.

### 6.5 Fibrotic remodeling: a notable evidence gap

Fibrotic remodeling is a defining DCM endpoint, yet the USP-fibrosis evidence base remains far thinner than that of the four pathological modules above. USP18-Notch1-AKT signaling (Section 3.5) contributes cytoprotection but does not directly target fibroblast activation (Cheng et al., 2025). No maladaptive USP has been demonstrated to drive DCM fibrosis directly, although USP24 and USP14 may contribute indirectly through their upstream inflammatory and ferroptotic effects (Wu et al., 2024; Wang et al., 2026). Thus, fibrosis remains one of the least substrate-resolved USP programs in non-ischemic DCM. Direct identification of fibroblast-specific USP-substrate axes represents an important priority for future investigation.

## 7 Cell type- and stage-resolved view of USP function in DCM

### 7.1 Cardiomyocyte versus non-myocyte compartments

Current DCM-USP evidence remains predominantly cardiomyocyte-centered, but it is no longer exclusively so. Cardiomyocyte studies have characterized USP7, USP8, USP13, USP14, USP18, USP20, USP24, USP28 and USP38 across metabolic, inflammatory, ferroptotic and autophagic pathways. This evidence asymmetry has important translational implications. The clearest illustration is USP13: cardiomyocyte-protective in DCM through K63-disassembly on NLRP3 (Xu D. et al., 2026), but macrophage-amplifying through K48-cleavage on the same substrate (Li et al., 2025). Systemic USP13 modulation would therefore engage opposing effects depending on which compartment dominates the diabetic myocardial inflammatory milieu. USP20 displays a less divergent pattern, with protective effects reported through STING clearance in cardiomyocytes (Zhou et al., 2026), and RIPK1 deubiquitination in vascular smooth muscle cells (Jean-Charles et al., 2018). Nevertheless, evidence from other cardiac cell populations remains insufficient to predict the net effect of systemic USP20 modulation. Importantly, recent endothelial-specific evidence identifies USP33 as a regulator of ATG7-dependent FIS1 clearance, mitochondrial fission and cardiac microvascular function in DCM (Chen et al., 2026). This finding establishes endothelial USP biology as a direct component of the DCM proteostatic network and indicates that cardiomyocyte-centered mechanisms cannot be extrapolated to the whole heart without considering cell-specific substrate programs.

### 7.2 A hypothesis-generating temporal view of USP function across DCM progression

DCM progresses from metabolic dysfunction toward inflammatory amplification, cell death, fibrosis and heart failure. Current cross-sectional studies suggest that different USP-substrate axes may become relevant at different pathological stages; however, no longitudinal study has established temporal dominance of individual USPs. Metabolic axes such as USP28-PPARα/Mfn2, USP7-PGC1β and USP38-ACAD11 may be particularly relevant to early metabolic remodeling, whereas USP13-NLRP3, USP20-STING, USP24-associated signaling and USP14-VDAC1 are linked to inflammatory or cell-death phenotypes. This organization should be regarded as a conceptual framework rather than evidence for stage-specific treatment. Longitudinal and temporally controlled perturbation studies are required to determine whether USP substrate engagement changes during DCM progression.

## 8 Translational considerations and future directions for USP-substrate modulation

### 8.1 Pharmacological modulation of injurious USP-substrate axes

Pharmacological modulation of USPs remains largely unexplored in DCM. USP7 provides the clearest example of this limitation. Although inhibition of the USP7-PGC1β axis may reduce maladaptive metabolic remodeling (Yan et al., 2024), the USP7-NRF2 study indicates that the same intervention can destabilize NRF2 and impair empagliflozin-mediated ferroptosis resistance (Cui et al., 2026). Thus, global USP7 inhibition could suppress an injurious substrate pathway while simultaneously impairing an adaptive one. Selective USP7 inhibitors developed mainly in oncology, including FT827 and related compounds, should therefore be regarded as mechanistic probes rather than candidate DCM therapies (Turnbull et al., 2017). A more rational future strategy would be to disrupt the disease-relevant USP7-PGC1β interaction while preserving USP7-NRF2 signaling.

A similar caution applies to USP14. IU1 provides an experimental tool for probing USP14-dependent proteasomal regulation (Lee et al., 2010), whereas b-AP15 should not be considered a selective USP14 inhibitor because it also inhibits UCHL5 and induces broader proteotoxic and mitochondrial stress (D'Arcy et al., 2011; Zhang et al., 2018). Although the USP14-VDAC1, USP24-associated and USP38-ACAD11 axes provide mechanistic rationales for future pharmacological investigation, myocardial exposure, substrate selectivity, efficacy and long-term safety remain to be established (Wu et al., 2024; Xiao et al., 2025; Wang et al., 2026; Wu et al., 2026).

### 8.2 Experimental restoration of protective USP-substrate axes

Genetic studies suggest that restoration of several protective USP-substrate axes can ameliorate experimental diabetic myocardial injury. Cardiomyocyte-directed USP28 restoration preserves PPARα-Mfn2-dependent mitochondrial integrity (Xie et al., 2024), USP13 suppresses NLRP3-dependent pyroptosis (Xu D. et al., 2026), and USP20 facilitates p62-dependent clearance of STING (Zhou et al., 2026). In addition, H_2_S-dependent USP8 activation promotes Parkin-mediated mitophagy, whereas endothelial USP33 stabilizes ATG7 and limits FIS1-dependent mitochondrial fission (Sun et al., 2020; Chen et al., 2026). These pathways therefore support further mechanistic investigation rather than immediate therapeutic extrapolation.

These findings establish causal and mechanistic proof-of-concept, but they do not yet validate USP restoration as a therapeutic strategy. Future approaches may require cardiac- and cell type-selective delivery or substrate-selective protein stabilization, with careful assessment of dose control, tissue specificity and long-term safety.

### 8.3 Integration with current cardiometabolic therapy and priorities for future validation

The USP7-NRF2 study further suggests that established cardiometabolic therapies may modify USP-substrate engagement rather than simply alter USP abundance (Cui et al., 2026). Future studies should therefore determine whether SGLT2 inhibitors, GLP-1 receptor agonists and other cardiometabolic therapies reshape myocardial ubiquitination and deubiquitination networks. Before USP-directed modulation can progress toward therapeutic development, several requirements remain: validation of disease-relevant USP-substrate axes in human diabetic myocardium, demonstration of myocardial and cell type-specific target engagement, definition of substrate selectivity, quantitative pharmacokinetic/pharmacodynamic assessment and long-term safety evaluation. At present, USP modulation should therefore be considered a framework for testable translational hypotheses rather than an established treatment strategy for DCM. These potential translational directions and the prerequisites for their future evaluation are summarized in Figure 3.

**FIGURE 3:**
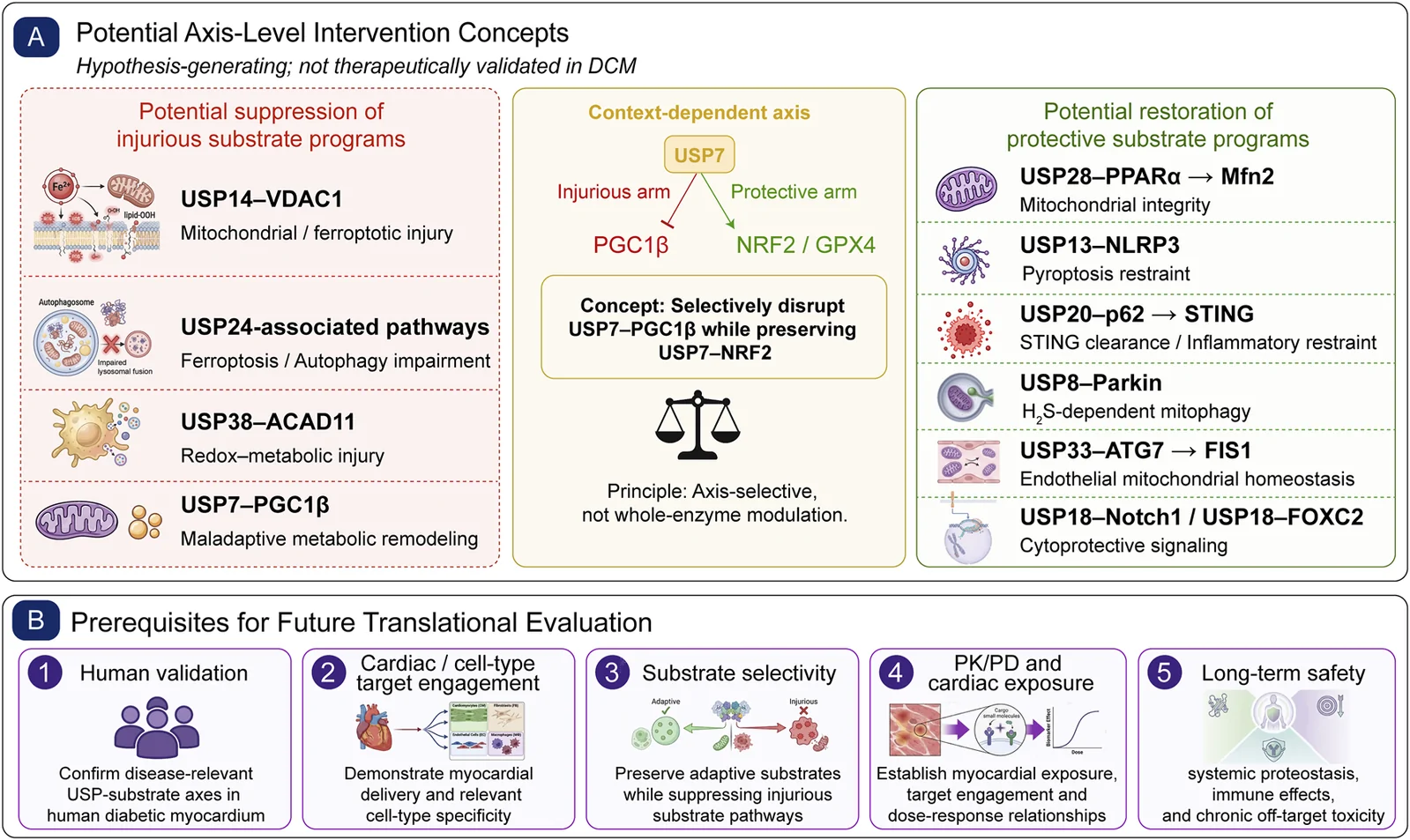
Potential translational directions for USP-substrate modulation in diabetic cardiomyopathy. **(A)** Hypothesis-generating axis-level intervention concepts based on currently reported USP-substrate relationships in diabetic cardiomyopathy (DCM). Predominantly injurious programs include USP14-VDAC1, USP24-associated ferroptotic and autophagy-disruptive signaling, USP38-ACAD11 and USP7-PGC1β. USP7 is highlighted as a context-dependent regulator because USP7-PGC1β promotes maladaptive metabolic remodeling, whereas USP7-NRF2 supports GPX4-dependent ferroptosis resistance, illustrating the rationale for substrate-selective rather than whole-enzyme modulation. Potentially protective programs include USP28-PPARα/Mfn2, USP13-NLRP3, USP20-p62/STING, H_2_S-dependent USP8-Parkin signaling, endothelial USP33-ATG7/FIS1 and USP18-associated Notch1/FOXC2 signaling. These intervention concepts remain preclinical and should not be interpreted as therapeutically validated strategies in DCM. **(B)** Key prerequisites for future translational evaluation, including validation of disease-relevant USP-substrate axes in human diabetic myocardium, demonstration of cardiac and cell type-specific target engagement, establishment of substrate selectivity, characterization of pharmacokinetic/pharmacodynamic properties and myocardial exposure, and assessment of long-term systemic and cardiac safety. Green denotes predominantly protective axes, red denotes predominantly injurious axes, and amber denotes context-dependent or bidirectional regulation. PK, pharmacokinetics; PD, pharmacodynamics; DCM, diabetic cardiomyopathy.

## 9 Challenges and future perspectives

Current DCM-USP findings derive almost entirely from STZ-induced diabetes, db/db mice, HFD/STZ models or high glucose/high lipid-treated cardiomyocytes. These systems are useful for mechanistic discovery, but they cannot fully recapitulate human DCM, which develops in the setting of ageing, obesity, hypertension, coronary artery disease, chronic kidney disease and heterogeneous cardiometabolic therapies. Therefore, the clearest priority is to determine whether the USP-substrate axes defined in experimental models are also present in human diabetic hearts (McCallinhart et al., 2025).

Recent human and omics-based studies provide important resources for this next step. Human myocardial multi-omics profiling of diabetic cardiomyopathy has revealed coordinated dysregulation of lipid metabolism, mitophagy and extracellular matrix remodeling in left ventricular tissues from patients with DCM, non-diabetic cardiomyopathy and healthy donors (Lu et al., 2026). This type of transcriptomic, proteomic and metabolomic dataset can be mined to test whether USP-related proteostatic modules correlate with human metabolic remodeling, mitochondrial quality control and fibrosis. In parallel, single-cell and spatial transcriptomic approaches are beginning to define cell type-specific and spatially resolved cardiac remodeling programs in diabetic or failing hearts (Koenig et al., 2022; McCallinhart et al., 2025), offering a framework for mapping USP expression across cardiomyocytes, fibroblasts, endothelial cells and immune cells. However, transcriptomic or proteomic abundance alone cannot establish USP function. USP mRNA or protein abundance does not necessarily define catalytic activity, substrate engagement or ubiquitin-chain preference (Mevissen and Komander, 2017; Rossio et al., 2024). Future studies should therefore combine human tissue datasets with activity-based DUB probes, chemical proteomics, ubiquitinomics, linkage-specific ubiquitin assays, IP-MS, proximity labeling and CRISPR-based perturbation. These technologies remain underused in diabetic myocardium but will be essential for distinguishing catalytically active disease-associated USPs from passive expression changes (Rossio et al., 2024).

A second limitation is the predominantly single-axis design of current studies. Individual USPs can regulate multiple substrates, while a single substrate may be controlled by several DUBs and competing E3 ligases; moreover, biological outcome depends on ubiquitin-chain topology and cellular context (Trulsson et al., 2022). Thus, future work should move from isolated USP-substrate pairs toward causal proteostatic networks. Proximity labeling, ubiquitin-remnant profiling, interaction proteomics and combinatorial CRISPR perturbation may help define how DUB-substrate-E3 relationships are reorganized during diabetic stress and identify the interactions most relevant to disease progression.

Several specific questions now warrant priority. First, how USP7 substrate engagement is redirected between PGC1β-driven metabolic remodeling and NRF2-dependent ferroptosis resistance remains unknown. Second, the USP33 study establishes direct endothelial USP biology in DCM, but comparable substrate-resolved evidence in fibroblasts and immune cells remains sparse. Resolving these substrate- and cell type-specific networks is likely to be more informative than searching for additional enzymes within a fixed protective/maladaptive classification. More broadly, human validation, cell type-specific target engagement and substrate-selective modulation should precede therapeutic extrapolation. Addressing these questions will be more informative than simply expanding the catalogue of USPs associated with DCM.

## 10 Conclusion

USP-dependent deubiquitination represents an important but highly context-dependent layer of diabetic myocardial proteostasis. Current evidence identifies USP-substrate axes that predominantly buffer diabetic myocardial stress, axes that predominantly amplify injury and bidirectional regulation exemplified by USP7, whose effects diverge between PGC1β-mediated metabolic remodeling and NRF2-dependent ferroptosis resistance. The addition of USP8-Parkin and endothelial USP33-ATG7/FIS1 further expands the DCM-USP landscape from cardiomyocyte-centered signaling toward mitophagy and cardiac microvascular proteostasis.

These findings argue against assigning fixed protective or maladaptive identities to individual USP enzymes. Future studies should instead define substrate engagement, catalytic activity, cardiac cell type and treatment context in human diabetic myocardium. Such an axis-centered framework provides a more rigorous basis for understanding proteostatic maladaptation and for developing substrate-selective, cell type-specific USP modulation in DCM.

## Glossary

ACAD11: Acyl-CoA dehydrogenase family member 11
AGEs: Advanced glycation end-products
AKT: Protein kinase B
AAV9: Adeno-associated virus serotype 9
ATG7: Autophagy-related protein 7
CAD: Coronary artery disease
cGAS: Cyclic GMP-AMP synthase
CKD: Chronic kidney disease
DCM: Diabetic cardiomyopathy
DUB: Deubiquitinating enzyme
ECM: Extracellular matrix
ER: Endoplasmic reticulum
FAO: Fatty acid oxidation
FIS1: Mitochondrial fission 1 protein
FOXC2: Forkhead box C2
GLP-1: Glucagon-like peptide-1
GPX4: Glutathione peroxidase 4
GSDMD: Gasdermin D
HFD/STZ: High-fat diet/streptozotocin
IL: Interleukin
IP-MS: Immunoprecipitation-mass spectrometry
Mfn2: Mitofusin 2
M1: Linear methionine 1-linked ubiquitin chain
NF-κB: Nuclear factor kappa B
NLRP3: NOD-, LRR- and pyrin domain-containing protein 3
NRF2: Nuclear factor erythroid 2-related factor 2
PGC1β: Peroxisome proliferator-activated receptor gamma coactivator 1 beta
PPARα: Peroxisome proliferator-activated receptor alpha
RIPK1: Receptor-interacting serine/threonine-protein kinase 1
ROS: Reactive oxygen species
SGLT2: Sodium-glucose cotransporter 2
STING: Stimulator of interferon genes
STAT1: Signal transducer and activator of transcription 1
TGF-β: Transforming growth factor beta
TRAF6: Tumor necrosis factor receptor-associated factor 6
UPS: Ubiquitin-proteasome system
USP: Ubiquitin-specific protease
